# Mupirocin-resistant *Staphylococcus aureus* in Africa: a systematic review and meta-analysis

**DOI:** 10.1186/s13756-018-0382-5

**Published:** 2018-08-15

**Authors:** Adebayo O. Shittu, Mamadou Kaba, Shima M. Abdulgader, Yewande O. Ajao, Mujibat O. Abiola, Ayodele O. Olatimehin

**Affiliations:** 1 Department of Microbiology, Obafemi Awolowo University, Ile-Ife, Osun State 22005 Nigeria; 2 Division of Medical Microbiology, Department of Pathology, Faculty of Health Sciences, University of Cape Town, Cape Town, South Africa; 3 Institute of Infectious Disease and Molecular Medicine, Faculty of Health Sciences, University of Cape Town, Cape Town, South Africa

**Keywords:** Africa, Prevalence, Meta-analysis, Mupirocin, *Staphylococcus aureus*, Systematic review

## Abstract

**Background:**

Mupirocin is widely used for nasal decolonization of *Staphylococcus aureus* to prevent subsequent staphylococcal infection in patients and healthcare personnel. However, the prolonged and unrestricted use has led to the emergence of mupirocin-resistant (mupR) *S. aureus*. The aim of this systematic review was to investigate the prevalence, phenotypic and molecular characteristics, and geographic spread of mupR *S. aureus* in Africa.

**Methods:**

We examined five electronic databases (EBSCOhost, Google Scholar, ISI Web of Science, MEDLINE, and Scopus) for relevant English articles on screening for mupR *S. aureus* from various samples in Africa. In addition, we performed random effects meta-analysis of proportions to determine the pooled prevalence of mupR *S. aureus* in Africa. The search was conducted until 3 August 2016.

**Results:**

We identified 43 eligible studies of which 11 (26%) were obtained only through Google Scholar. Most of the eligible studies (28/43; 65%) were conducted in Nigeria (10/43; 23%), Egypt (7/43; 16%), South Africa (6/43; 14%) and Tunisia (5/43; 12%). Overall, screening for mupR *S. aureus* was described in only 12 of 54 (22%) African countries. The disk diffusion method was the widely used technique (67%; 29/43) for the detection of mupR *S. aureus* in Africa. The *mupA*-positive *S. aureus* isolates were identified in five studies conducted in Egypt (*n* = 2), South Africa (*n* = 2), and Nigeria (*n* = 1). Low-level resistance (LmupR) and high-level resistance (HmupR) were both reported in six human studies from South Africa (*n* = 3), Egypt (*n* = 2) and Libya (*n* = 1). Data on mupR-MRSA was available in 11 studies from five countries, including Egypt, Ghana, Libya, Nigeria and South Africa. The pooled prevalence (based on 11 human studies) of mupR *S. aureus* in Africa was 14% (95% CI =6.8 to 23.2%). The proportion of *mupA*-positive *S. aureus* in Africa ranged between 0.5 and 8%. Furthermore, the frequency of *S. aureus* isolates that exhibited LmupR, HmupR and mupR-MRSA in Africa were 4 and 47%, 0.5 and 38%, 5 and 50%, respectively.

**Conclusions:**

The prevalence of mupR *S. aureus* in Africa (14%) is worrisome and there is a need for data on administration and use of mupirocin. The disk diffusion method which is widely utilized in Africa could be an important method for the screening and identification of mupR *S. aureus*. Moreover, we advocate for surveillance studies with appropriate guidelines for screening mupR *S. aureus* in Africa.

## Background

*Staphylococcus aureus* is a well-recognized human pathogen that is implicated in a wide array of superficial, invasive and toxigenic infections [1]. Meta-analyses of published studies have provided evidence that *S. aureus* nasal carriage is an important risk factor for subsequent infection among patients with surgical site infections and atopic dermatitis [2, 3]. Other high-risk groups include patients colonized with methicillin-resistant *Staphylococcus aureus* (MRSA) undergoing dialysis, and patients admitted in the intensive care unit [4, 5]. Consequently, infection prevention strategies such as nasal decolonization are employed to minimize the occurrence of staphylococcal infection and reduce the risk of transmission in healthcare settings [6, 7]. Mupirocin (2%) nasal ointment alone or in combination with 4% chlorhexidine (CHG) based body wash is considered as the main decolonization strategy for *S. aureus* carriage [8, 9]. Mupirocin is a naturally occurring antibiotic produced by *Pseudomonas fluorescens* that interferes with protein synthesis by competitive inhibition of the bacterial isoleucyl-tRNA synthetase (IRS) [10, 11]. It gained prominence in the mid-1990s for the eradication of *S. aureus* nasal carriage due to its effectiveness, safety and cost [12].

Mupirocin-resistant (mupR) *S. aureus* was first reported in the United Kingdom in 1987 [13]. Since then, it has been reported in several countries worldwide [14–17]. The emergence of mupR *S. aureus* has been associated with unrestricted policies and use of mupirocin for long periods in health care settings [8, 18]. Decolonization failure in patients with *S. aureus* carriage is associated with high-level mupirocin resistance (HmupR - minimum inhibitory concentration [MIC]: ≥512 μg/ml), while that of low-level mupirocin resistance (LmupR – MIC: 8-64 μg/ml) is still unclear [7, 19]. LmupR is mediated through point mutation (largely V588F and V631F) in the native isoleucyl-tRNA synthetase (*ileS*) gene [20]. In contrast, HmupR is mainly attributed to the acquisition of plasmids with the *mupA* (or *ileS2*) gene encoding an additional IRS with no affinity for mupirocin [11, 21]. Another determinant for HmupR is the acquisition of a plasmid-mediated *mupB* gene [22].

There is no data summarizing reports on screening, prevalence, characterization, and geographic spread of mupR *S. aureus* in Africa. This systematic review evaluated published articles that assessed for mupirocin resistance in African *S. aureus* isolates. The findings from this systematic review highlight the need to develop an early warning system, including harmonized strategies for the prompt screening and identification of mupR *S. aureus* in Africa.

## Methods

### Literature search strategy

The relevant English articles from human and animal investigations were retrieved by three authors (YA, SA, and AS) from five electronic databases (EBSCOhost, Google Scholar, ISI Web of Science, MEDLINE, and Scopus). The search terms for each database are reported in Table 1. The literature search was concluded on 3 August 2016.

**Table 1:** Keywords used to identify eligible studies available in five biomedical databases

Database	Search period	Search strategy
MEDLINE via PubMed EBSCOhost via Academic Search premier, Africa-Wide information and CINAHL	1974 - August 2016 1982 - August 2016	(Staphylococcus aureus OR S. aureus) AND (Mupirocin) AND (Algeria OR Angola OR Benin OR Botswana OR Burkina Faso OR “Burkina Faso” OR Burkina Fasso OR Upper Volta OR “Upper Volta” OR Burundi OR Cameroon OR Cape Verde OR “Cape Verde” OR Central African Republic OR Chad OR Comoros OR “Iles Comores” OR Iles Comores OR Comoro Islands OR “Comoro Islands” OR Congo OR Democratic Republic Congo OR “Democratic Republic of the Congo” OR Zaire OR Djibouti OR Egypt OR Equatorial Guinea OR “Equatorial Guinea” OR Eritrea OR Ethiopia OR Gabon OR Gambia OR Ghana OR Guinea OR Guinea Bissau OR “Guinea Bissau” OR Ivory Coast OR “Ivory Coast” OR Cote d’Ivoire OR “Cote d’Ivoire” OR Kenya OR Lesotho OR Liberia OR Libya OR Libia OR Jamahiriya OR Jamahiryia OR Madagascar OR Malawi OR Mali OR Mauritania OR Mauritius OR Ile Maurice OR “Ile Maurice” OR Morocco OR Mozambique OR Moçambique OR Namibia OR Niger OR Nigeria OR Rwanda OR Sao Tome OR “Sao Tome” OR Senegal OR Seychelles OR Sierra Leone OR “Sierra Leone” OR Somalia OR South Africa OR “South Africa” OR Sudan OR South Sudan OR “South Sudan” OR Swaziland OR Tanzania OR Tanganyika OR Zanzibar OR Togo OR Tunisia OR Uganda OR Western Sahara OR “Western Sahara” OR Zambia OR Zimbabwe OR Africa OR Africa* OR Southern Africa OR West Africa OR Western Africa OR Eastern Africa OR East Africa OR North Africa OR Northern Africa OR Central Africa OR Sub Saharan Africa OR Subsaharan Africa OR Sub-Saharan Africa) NOT (Guinea pig* OR “Guinea pig*” OR Aspergillus niger OR “Aspergillus niger” OR Europe* OR America* OR Asia*)
ISI Web of Science	1950 - August 2016	
Scopus from SciVerse	1982 - August 2016	(Staphylococcus aureus OR S. aureus) AND (Mupirocin) AND (Africa)^a^
Google Scholar**		(Staphylococcus aureus OR S. aureus) AND (Mupirocin) AND (Name of each African country) Examples (Staphylococcus aureus OR S. aureus) AND (Mupirocin) AND (Algeria) (Staphylococcus aureus OR S. aureus) AND (Mupirocin) AND (Zimbabwe)

^a^The African countries were manually selected (as recommended by Scopus database) to exclude studies from other continents

**The Google Scholar search was conducted between July-September 2015

### Eligible article identification

The identification of the eligible articles was conducted according to the guidelines for preferred reporting items for systematic reviews and meta-analyses (PRISMA) [23]. We defined an eligible article as a peer-reviewed publication that (i) included mupirocin in the antibiotic susceptibility testing of *S. aureus* isolates, and (ii) employed phenotypic ((disc diffusion, E-test, minimum inhibitory concentration (MIC), VITEK and other automated methods)), and/or molecular ((conventional or real-time polymerase chain reaction (PCR)) techniques. International multicentre studies that included African countries were also eligible for inclusion.

### Data extraction and analysis

**Fig. 1:**
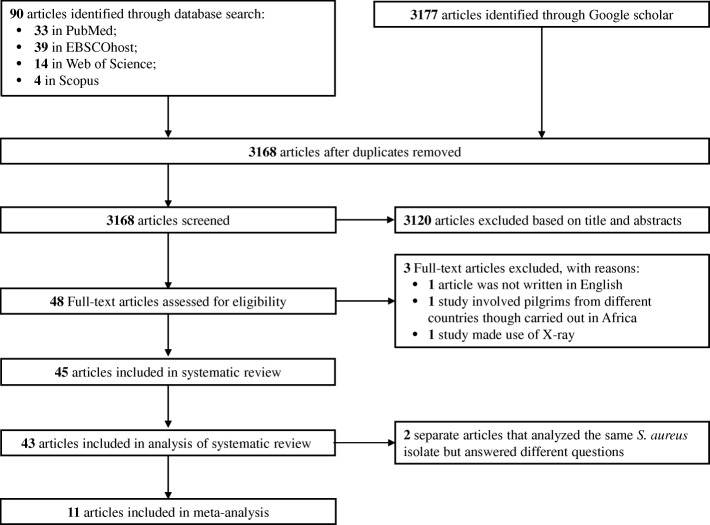
The Preferred Reporting Items for Systematic Review and Meta-analysis flow diagram

The relevant data were extracted from each of the eligible articles included in this systematic review. A study that analysed *S. aureus* isolates from another investigation but answered a different research question were both considered as one study (Table 2). We performed three levels of analysis (Fig. 1). First, to understand the characteristics and geographic spread of mupR *S. aureus* in Africa, studies that included mupirocin in the antibiotic susceptibility testing and employed phenotypic and/or molecular techniques were identified. Secondly, the prevalence of *S. aureus* with the *mupA* gene, isolates that expressed LmupR and HmupR, and mupR-MRSA in Africa were derived from each eligible study as follows:

$$ MupA\hbox{-} \mathrm{positive}\ S.\kern0.5em aureus=\frac{\mathrm{Number}\ \mathrm{of}\  MupA\hbox{-} \mathrm{positive}\ S.\kern0.5em aureus\ \mathrm{isolates}}{\mathrm{Total}\ \mathrm{number}\ \mathrm{of}\ \mathrm{isolates}\ \mathrm{screened}\ \mathrm{with}\ \mathrm{mupirocin}} $$

$$ S.\kern0.5em aureus\ \mathrm{that}\ \mathrm{expressed}\ \mathrm{LmupR}=\frac{\mathrm{Number}\ \mathrm{of}\ S.\kern0.5em aureus\ \mathrm{isolates}\ \mathrm{with}\ \mathrm{LmupR}}{\mathrm{Total}\ \mathrm{number}\ \mathrm{of}\ \mathrm{isolates}\ \mathrm{screened}\ \mathrm{with}\ \mathrm{mupirocin}} $$

$$ S.\kern0.5em aureus\ \mathrm{that}\ \mathrm{expressed}\ \mathrm{HmupR}=\frac{\mathrm{Number}\ \mathrm{of}\ S.\kern0.5em aureus\ \mathrm{isolates}\ \mathrm{with}\ \mathrm{HmupR}}{\mathrm{Total}\ \mathrm{number}\ \mathrm{of}\ \mathrm{isolates}\ \mathrm{screened}\ \mathrm{with}\ \mathrm{mupirocin}} $$

$$ \mathrm{MupR}\hbox{-} \mathrm{MRSA}=\frac{\mathrm{Number}\ \mathrm{of}\ \mathrm{mupR}\hbox{-} \mathrm{MRSA}\ \mathrm{isolates}}{\mathrm{Total}\ \mathrm{number}\ \mathrm{of}\ \mathrm{isolates}\ \mathrm{screened}\ \mathrm{with}\ \mathrm{mupirocin}} $$

**Table 2:** Characteristics of the 43 eligible studies on screening for mupirocin resistance in *Staphylococcus aureus* from various sources in Africa

Region	Country	Study Period	Setting	Sample		Method for testing resistance to mupirocin	Guideline (year of publication)	Published reports for detection of mupR *S. aureus*	Number of *S. aureus* isolates screened with mupirocin	Mupirocin resistant isolates				Reference
				Source	Type					Number (%)	Number MRSA (%)	Number LmupR/HmupR	Number *mupA* gene + LMupR/HmupR (Method)	
North Africa	Algeria	2005–2007	C & H	Human	Pus, venous catheter, tracheal aspirate, punction fluid, blood, urine	Disk diffusion VITEK-2	CLSI (NA)	–	19	0 (0)	0 (0)	–	–	[47]
	Egypt	2005–2006	C & H	Human	NA	Disk diffusion	NCCLS (2003)	–	64	0 (0)	0 (0)	–	–	[28]
	Egypt	2008–2009	C & H	Human	Skin and soft tissue, post-operative wound swab	Disk diffusion	CLSI (2007)	–	386	1 (0.3)	NA	NA	–	[29]
	Egypt	2007–2008	C	Human	Pus, sputum, catheter, blood, urine, wound abscess	Broth dilution	CLSI (2005)	–	21	0 (0)	0 (0)	–	–	[58]
	Egypt	2010	H	Human	Sputum, blood, catheter, traumatic wound, urine	E-test	–	Kresken et al., (2004)	86	30 (34.9)	30 (34.9)	25/5	2/3 (PCR)	[30]
	Egypt	2012	H	Human	Wound discharge, blood, body fluid aspirate, urine, faeces, sputum, nasal, throat, ear and genital swab	Disk diffusion Agar dilution	CLSI (2007)	–	150	0 (0)	0 (0)	–	–	[40]
	Egypt	2012–2013	H	Human	Nasal swab	Disk diffusion	CLSI (2011)	–	39	3 (7.7)	3 (3.7)	NA	–	[31]
	Egypt	2013–2015	H	Human	Pus & Wound swab	Agar dilution	CLSI (2011)	–	73	13 (17.8)	13 (17.8)	5/8	0/6 (PCR)	[52]
	Libya	NA	H	Human	Skin swab	Disk diffusion	NA	–	40	0 (0)	NA	–	–	[61]
	Libya	2008–2009	H	Human & Environment	NA	Disk diffusion	BSAC (2008)	–	86	13 (15.1)	13 (8.1)	NA	–	[56]
	Libya	2009	H	Human	Nasal swab	Disk diffusion Agar dilution	BSAC (2008)	–	109	5 (4.6)	5 (4.6)	4/1	–	[57]
	Morocco	2008-	H	Human	Nasal swab	Disk diffusion	CA-SFM (2007)	–	81	0 (0)	0 (0)	–	–	[62]
	Tunisia	2008–2009	C	Human	Nasal swab	Disk diffusion	CLSI (2008)	–	55	0 (0)	0 (0)	–	–	[41]
	Tunisia	2003–2005	C	Human	Pus, blood, articular puncture, venous catheter	Phoenix Automated Microbiology System	CA-SFM (2006)	–	64	NA	NA	–	–	[59]
	Tunisia	2013	H	Human	Wound abscess	Disk diffusion	CA-SFM (2013)	–	8	NA	NA	–	–	[60]
	Tunisia	2010	C	Animal (Sheep)	Nasal swab	Disk diffusion	CLSI (2010)	–	73	0 (0)	0 (0)	–	–	[42]
	Tunisia	2010	C	Animal (Donkeys)	Nasal swab	Disk diffusion	CLSI (2010)	–	50	0 (0)	0 (0)	–	–	[43]
West Africa	Ghana	2011–2012	H	Human	Nasal swab	Disk diffusion	EUCAST (2012)	–	105	1 (0.9)	0 (0)	0/1	–	[54]
	Ghana	2011–2012	C	Human	Nasal swab	Disk diffusion	EUCAST (2012)	–	124	0 (0)	0 (0)	–	–	[67]
	Ghana	2010–2013	C & H	Human	NA	Broth microdilution	EUCAST (NA)	–	30	4 (13.3)	4 (13.3)	4/0	0/0 (DNA microarray)	[55]
	Ghana	2012–2013	C	Human	Nasal & Wound swab	VITEK-2	EUCAST (NA)	–	91	0 (0)	0 (0)	–	–	[68]
	Nigeria*	NA	NA	Human	NA	Disk diffusion	NA	–	1	0 (0)	0 (0)	–	–	[80]
	Nigeria*	2002–2004	H	Human	Wound, blood, ear, eye, urine	Disk diffusion	–	Udo et al., (1999)	200	1 (0.5)	0 (0)	0/1	0/1 (PCR)	[53]
	Nigeria	2006	C	Human	Nasal swab	Disk diffusion	CLSI (2005)	–	101	12 (11.9)	NA	NA	–	[44]
	Nigeria	2007	H	Human	NA	Disk diffusion	CLSI (NA)	–	96	0 (0)	0 (0)	–	–	[48]
	Nigeria*	NA	H	Human	Wound swab, blood, urine, endotracheal aspirate	Disk diffusion E-test	NCCLS (2003)	–	1	1	0 (0)	0/1	0/1 (PCR)	[45]
	Nigeria	2009	H	Human	Wound, sputum, semen, nasal swab	Broth microdilution	DIN 58940 (2004)	–	68	0 (0)	0 (0)	–	–	[63]
	Nigeria	2010	H	Human	NA	VITEK-2	–	–	51	0 (0)	0 (0)	–	–	[64]
	Nigeria	2009–2011	H	Human	Aspirate, blood, ear, eye, vaginal discharge, sputum, wounds, urine, nasal swab	Disk diffusion	CLSI (NA)	–	62	0 (0)	0 (0)	–	–	[49]
	Nigeria	2010–2011	H	Human	NA	VITEK-2	EUCAST (NA)	–	290	0 (0)	0 (0)	–	–	[65]
	Nigeria	2008–2010	C	Animal (Bats)	Faecal swab	Disk diffusion	–	Udo et al., (1999)	107	0 (0)	0 (0)	–	–	[66]
	Nigeria	2006–2007	C & H	Animal (Bovine) & (Ovine)	Nasal & skin swab	Disk diffusion	–	Udo et al., (1999)	173	0 (0)	0 (0)	–	–	[35]
	Nigeria	2012	C	Human Animal	Nasal swab Milk	Disk diffusion	CLSI (2006)	–	10 Humans 77 Animals	33 (37.9)	NA	0/33	–	[36]
Central Africa	Gabon	2009	C & H	Human	Nasal, axillae, inguinal swab	VITEK-2	–	–	5	0 (0)	0 (0)	–	–	[69]
	São Tomé & Príncipe	2010–2012	H	Human	Nasal swab	Disk diffusion	BSAC (NA)	–	55	0 (0)	0 (0)	–	–	[70]
East Africa	Ethiopia	NA	H & R	Cockroach	Cockroach Body surface/Gut	Disk diffusion	–	Jorgenson et al., (1999)	17	17 (100)	NA	NA	–	[37]
	Kenya	2011	H	Human	Nasal and axillary skin swab	VITEK-2	CLSI (2012)	–	86	0 (0)	0 (0)	–	–	[71]
	Kenya	2011–2013	H	Human	Pus, blood, urine	VITEK-2	CLSI (2010)	–	731	0 (0)	0 (0)	–	–	[72]
	Kenya	NA	C	Animal (Camel)	Raw camel milk	Disk diffusion Broth microdilution	CLSI (2008)	–	47	0 (0)	0 (0)	–	–	[38]
South Africa	South Africa	1996	H	Human	Wound, urine, skin and blood	Disk diffusion	NCCLS (2000)	–	236	5 (2.1)	NA	NA	–	[46]
	South Africa**	2001–2003	H	Human	Wound, sputum, blood	Disk diffusion	–	Udo et al., (1999)	227	16 (7.0)	15 (6.6)	14/2	0/2 (PCR)	[50]
	South Africa	2005–2006	H	Human	Blood, pus & skin wound, cerebrospinal fluid	Disk diffusion E-test	–	Udo et al., (2006)	248	123 (49.6)	123 (49.6)	117/6	–	[32]
	South Africa**	NA	H	Human	Wound swab, blood, urine, endotracheal aspirate	Disk diffusion E-test	NCCLS (2003)	–	16	16 (100)	14 (87.5)	14/2	0/2 (PCR)	[45]
	South Africa	2013	H	Human	Tissue, blood, cerebrospinal fluid, wound swab	Disk diffusion VITEK-2	CLSI (2012)	–	997	277 (27.8)	NA	43/234	0/5 (Real time PCR)	[33]
	South Africa	2010–2012	H	Human	Blood	Microscan (MIC Panel Type 33)	CLSI (2015)	–	2709	236 (8.7)	202 (7.5)	NA	–	[51]
	South Africa	2009–2010	H	Human & Environment	Nasal & hand swab, dialysate fluid, surface swab, air samples	VITEK-2	–	–	13	4 (30.8)	4 (30.8)	0/4	–	[34]

KEY: mupR *S. aureus*: mupirocin resistant *Staphylococcus aureus*; *LmupR* low-level mupirocin resistance, *HmupR* high-level mupirocin resistance, *mupA* mupirocin resistance gene, *MIC* Minimum inhibitory concentration, *BSAC* British Society for Antimicrobial Chemotherapy, *CA-SFM* Comité de l’Antibiogramme de la Société Française de Microbiologie, *CLSI* Clinical and Laboratory Standards Institute, *DIN 58940* Deutsches Institut für Normung DIN 58940, *EUCAST* European Committee on Antimicrobial Susceptibility Testing, *NCCLS* National Committee for Clinical Laboratory Standards, *PCR* Polymerase Chain Reaction; − Not determined, *NA* Not available, *H* Hospital, *C* Community, *R* Restaurant

*Separate reports that analyzed the same isolates but answered different questions (considered as one single study) in Nigeria; **: Separate reports that analyzed the same isolates but answered different questions (considered as one single study) in South Africa.

Reference [45] is recorded in Nigeria and South Africa, but the isolates were derived from studies in Nigeria [53] and South Africa [50], respectively

Other published reports applied for the detection of mupR *S. aureus* in Africa

1. Jorgenson JH, Turnidge JD, Washington JA. Dilution and disc diffusion method. In: Murray PR, Baron EJ, Pfaller MA, Tenover FC, Yolken RH, editors. Manual of Clinical Microbiology, 7th edition. American Society for Microbiology, Washington DC, 1999. p. 1526–1543. Adapted from NCCLS: National Committee for Clinical Laboratory Standards 1997. Approved Standard M2-A6; National Committee for Clinical Laboratory Standards 1999. Approved Standard M100-S9.

2. Kresken M, Hafner D, Schmitz FJ, Wichelhaus TA. Prevalence of mupirocin resistance in clinical isolates of *Staphylococcus aureus* and *Staphylococcus epidermidis*. Results of the antimicrobial resistance surveillance study of the Paul-Ehrlich Society for Chemotherapy, 2001. Int J Antimicrob Agents, 2004, 23:577–81. The widely accepted breakpoints: ≤4 mg/l (susceptible), 8–256 mg/l (low-level resistance) and ≥ 512 mg/l (high-level resistance) was utilized in this study.

3. Udo EE, Farook VS, Mokadas EM, Jacob LE, Sanyal SC. Molecular fingerprinting of mupirocin-resistant methicillin-resistant *Staphylococcus aureus* from a burn unit. Int J Infect Dis, 1999,3:82–7. Growth within a 14-mm zone of inhibition with the 5 μg mupirocin disk detected low-level resistance, while growth to the edge of the 200 μg mupirocin disk indicated high-level resistance.

4. Udo EE, Al-Sweih N, Mokaddas E, Johny M, Dhar R, Gomaa HH, Al-Obaid I, Rotimi VO. Antibacterial resistance and their genetic location in MRSA isolated in Kuwait hospitals, 1994–2004. BMC Infect Dis, 2006;6:168. The widely accepted breakpoints:≤4 mg/l (susceptible), 8–256 mg/l (low-level resistance) and ≥ 512 mg/l (high-level resistance) was utilized in this study.

Thirdly, to estimate the prevalence of mupR *S. aureus* in humans, studies that employed at least one of the screening methods with defined breakpoint for mupirocin resistance were included in the meta-analysis. The StatsDirect statistical software version 3.0.165 (England: StatsDirectLtd.2016) was utilized to assess the heterogeneity of the eligible studies included in the meta-analysis (Cochran Q-test) [24], and to ascertain the inconsistency across the studies (I2 statistic) [25]. The random effects model was used to determine the pooled prevalence of mupR *S. aureus* in Africa. The criterion for statistical significance for heterogeneity was set at alpha = 0.05. The risk of publication bias was assessed and visualized by a Funnel plot [26, 27].

## Results

### Eligible studies from electronic database search

We identified 43 reports (Table 1) of which 34 studies investigated only human samples. The remaining nine studies assessed samples from only animals (*n* = 5), human and environmental sources (*n* = 2), human and animal sources (*n* = 1), and cockroaches (n = 1). Most of the eligible studies (32/43; 74%) were obtained from EBSCOhost, ISI Web of Science, MEDLINE, and Scopus. The remaining studies (11/43; 26%) were obtained only through Google Scholar and consisted of studies conducted in Egypt [28–31], South Africa [32–34], Nigeria [35, 36], Ethiopia [37] and Kenya [38].

### Screening and identification of mupR *S. aureus* in Africa

**Fig. 2:**
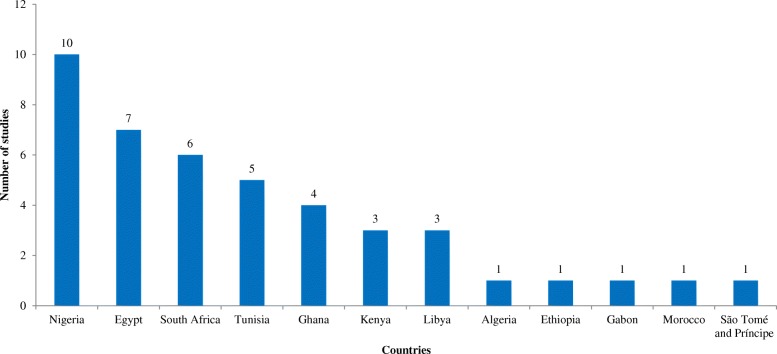
Studies on screening for mupirocin-resistant *Staphylococcus aureus* in Africa

Only 12 of the 54 (22%) African countries reported data on screening for mupR *S. aureus* (Fig. 2). The first published article indicated that mupirocin had been in use in Africa, at least from the late 1980s [39]. Most of these studies (28/43; 65%) were conducted in Nigeria (10/43; 23%), Egypt (7/43; 16%), South Africa (6/43; 14%) and Tunisia (5/43; 12%) (Fig. 2). MupR *S. aureus* was mainly identified through the disk diffusion method (29/43; 67%). The guidelines by the Clinical and Laboratory Standards Institute (CLSI), previously known as National Committee for Clinical Laboratory Standards (NCCLS), were broadly used in Africa (Table 2). However, a number of studies [28, 29, 31, 33, 36, 40–46] utilized the disk diffusion method with CLSI guidelines that had no zone diameter breakpoint for mupirocin. Moreover, some studies [47–49] did not provide information on the year of publication of the CLSI guidelines. MupR *S. aureus* was reported in six African countries including South Africa [32–34, 46, 50, 51], Egypt [29–31, 52], Nigeria [36, 44, 53], Ghana [54, 55], Libya [56, 57] and Ethiopia [37] (Fig. 2; Table 2). The *mupA*-positive *S. aureus* was detected in five studies from Egypt [30, 52], South Africa [33, 50] and Nigeria [53]. LmupR and HmupR were both reported in six human studies conducted in South Africa [32, 33, 50], Egypt [30, 52] and Libya [57]. The mupR-MRSA isolates were identified in South Africa [32, 34, 50, 51], Egypt [30, 31, 52], Libya [56, 57], Ghana [55] and Nigeria [36] (Table 3). MupR-MRSA was not reported from MRSA isolates recovered from studies conducted in Egypt [28, 58], Tunisia [59, 60] and Algeria [47].

**Table 3:** Prevalence of mupirocin-resistant *S. aureus* from various sources in Africa based on phenotypic and molecular methods

Mupirocin resistance	Country	Source	Number positive/Total tested (%)	Phenotypic						Molecular		Guidelines or reports				Reference
				Agar Dilution	Broth microdilution	Disk diffusion	E-test	Microscan system	VITEK	PCR	Micro array	BSAC	CLSI	EUCAST	Other reports	
*MupA*-positive *S. aureus*	Egypt	Human	5/86 (5.8)	–	–	–	√	–	–	√	–	–	–	–	√^a^	[30]
	Egypt	Human	6/73 (8.2)	√	–	–	–	–	–	√	–	–	√	–	–	[52]
	Nigeria	Human	1/200 (0.5)	–	–	√	–	–	–	√	–	–	–	–	√^b^	[53]
	South Africa	Human	2/227 (0.9)	–	–	√	–	–	–	√	–	–	–	–	√^b^	[50]
	South Africa	Human	NA	–	–	√	–	–	√	√	–	–	√	–	–	[33]
LmupR *S. aureus*	Egypt	Human	25/86 (29.1)	–	–	–	√	–	–	√	–	–	–	–	√^a^	[30]
	Egypt	Human	5/73 (6.8)	√	–	–	–	–	–	√	–	–	√	–	–	[52]
	Ghana	Human	4/30 (13.3)	–	√	–	–	–	–	–	√	–	–	√	–	[55]
	Libya	Human	4/109 (3.7)	√	–	√	–	–	–	–	–	√	–	–	–	[57]
	South Africa	Human	14/227 (6.2)	–	–	√	–	–	–	–	–	–	–	–	√^b^	[50]
	South Africa	Human	117/248 (47.2)	–	–	√	√	–	–	–	–	–	–	–	√^c^	[32]
	South Africa	Human	43/997 (4.3)	–	–	√	–	–	√	–	–	–	√	–	–	[33]
	South Africa	Human & Environment	4/13 (30.8)	–	–	–	–	–	√	–	–	–	–	–	–	[34]
HmupR *S. aureus*	Egypt	Human	5/86 (5.8)	–	–	–	√	–	–	√	–	–	–	–	√^a^	[30]
	Egypt	Human	8/73 (11)	√	–	–	–	–	–	√	–	–	√	–	–	[52]
	Ghana	Human	1/105 (1.0)	–	–	√	–	–	–	–	–	–	–	√	–	[54]
	Libya	Human	1/109 (0.9)	√	–	√	–	–	–	–	–	√	–	–	–	[57]
	Nigeria	Human	1/200 (0.5)	–	–	√	–	–	–	√	–	–	–	–	√^b^	[53]
	Nigeria	Human	12/101 (11.9)	–	–	√	–	–	–	–	–	–	√	–	–	[44]
	Nigeria	Human & Animal	33/87 (37.9)	–	–	√	–	–	–	–	–	–	√	–	–	[36]
	South Africa	Human	2/227 (0.9)	–	–	√	–	–	–	√	–	–	–	–	√^b^	[50]
	South Africa	Human	6/248 (2.4)	–	–	√	√	–	–	–	–	–	–	–	√^c^	[32]
	South Africa	Human	234/997 (23.5)	–	–	√	–	–	√	–	–	–	√	–	–	[33]
mupR-MRSA	Egypt	Human	30/86 (34.9)	–	–	–	√	–	–	√	–	–	–	–	√^a^	[30]
	Egypt	Human	3/39 (7.7)	–	–	√	–	–	–	–	–	–	√	–	–	[31]
	Egypt	Human	13/73 (17.8)	√	–	–	–	–	–	√	–	–	√	–	–	[52]
	Ghana	Human	4/30 (13.3)	–	√	–	–	–	–	–	√	–	–	√	–	[55]
	Libya	Human	13/86 (15.1)	–	–	√	–	–	–	–	–	√	–	–	–	[56]
	Libya	Human	5/109 (4.6)	√	–	√	–	–	–	–	–	√	–	–	–	[57]
	Nigeria	Human & Animal	33/87 (37.9)	–	–	√	–	–	–	–	–	–	√	–	–	[36]
	South Africa	Human	15/227 (6.6)	–	–	√	–	–	–	√	–	–	–	–	√^b^	[50]
	South Africa	Human	123/248 (49.6)	–	–	√	√	–	–	–	–	–	–	–	√^c^	[32]
	South Africa	Human	202/2709 (7.5)	–	–	–	–	√	–	–	–	–	√	√	–	[51]
	South Africa	Human & Environment	4/13 (30.8)	–	–	–	–	–	√	–	–	–	–	–	–	[34]

KEY: *BSAC* British Society for Antimicrobial Chemotherapy, *CLSI* Clinical and Laboratory Standards Institute, *EUCAST* European Committee on Antimicrobial Susceptibility Testing, *NA* Not Available, *PCR* Polymerase Chain Reaction, √: test was performed. -: test was not performed

^a^The widely accepted breakpoints: ≤4 mg/l (susceptible), 8–256 mg/l (low-level resistance) and ≥ 512 mg/l (high-level resistance) was utilized in this study: Kresken M, Hafner D, Schmitz FJ, Wichelhaus TA. Prevalence of mupirocin resistance in clinical isolates of *Staphylococcus aureus* and *Staphylococcus epidermidis*. Results of the antimicrobial resistance surveillance study of the Paul-Ehrlich Society for Chemotherapy, 2001. Int J Antimicrob Agents, 2004, 23:577–81. ^b^Growth within a 14-mm zone of inhibition with the 5 μg mupirocin disk detected low-level resistance, while growth to the edge of the 200 μg mupirocin disk indicated high-level resistance according to: Udo EE, Farook VS, Mokadas EM, Jacob LE, Sanyal SC. Molecular fingerprinting of mupirocin-resistant methicillin-resistant *Staphylococcus aureus* from a burn unit. Int J Infect Dis, 1999,3:82–7. ^c^The widely accepted breakpoints: ≤4 mg/l (susceptible), 8–256 mg/l (low-level resistance) and ≥ 512 mg/l (high-level resistance) was utilized in this study: Udo EE, Al-Sweih N, Mokaddas E, Johny M, Dhar R, Gomaa HH, Al-Obaid I, Rotimi VO. Antibacterial resistance and their genetic location in MRSA isolated in Kuwait hospitals, 1994–2004. BMC Infect Dis, 2006;6:168

**Fig. 3:**
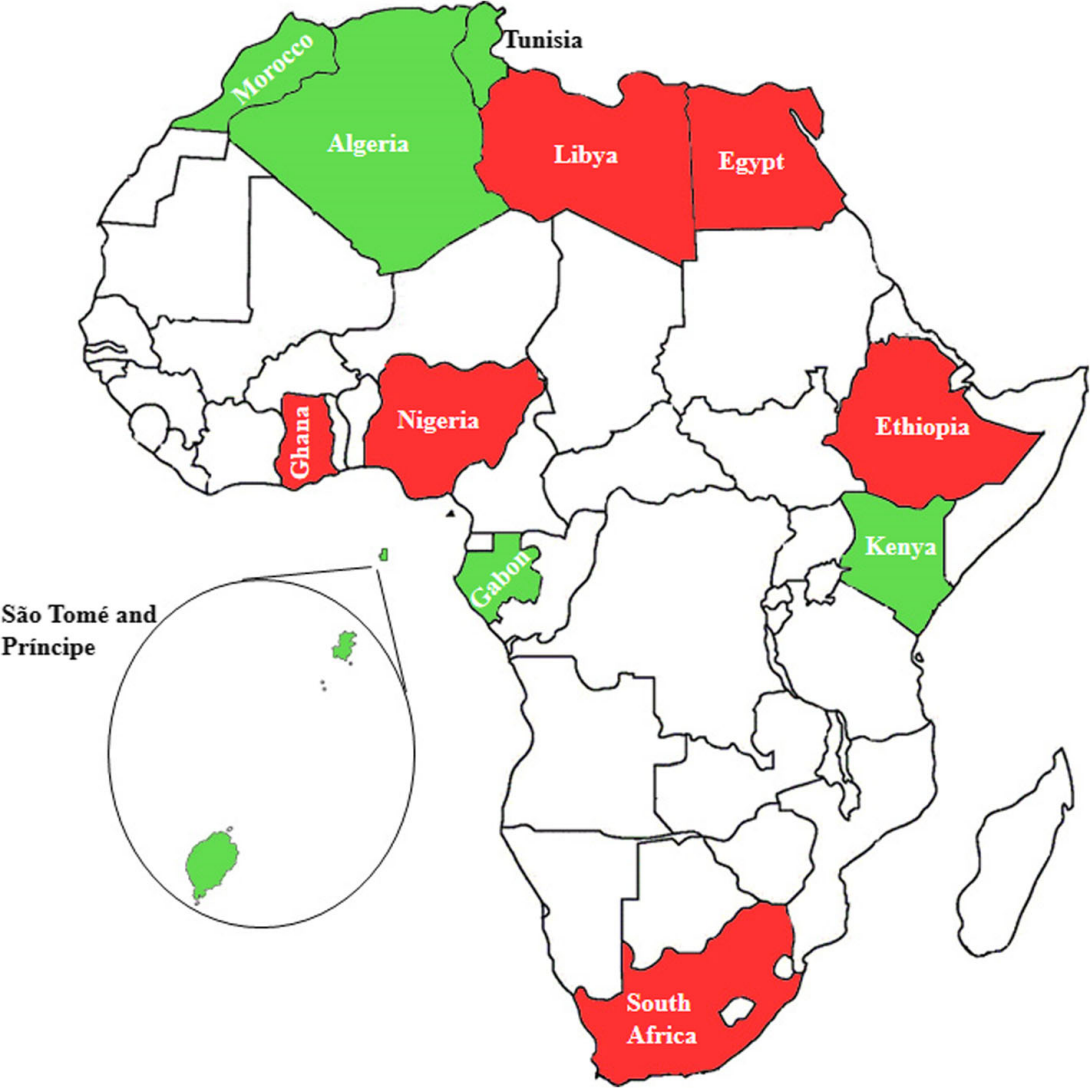
Geographic distribution of mupirocin-resistant (mupR) *Staphylococcus aureus* in Africa. Countries (in green) in which mupR *S. aureus* have been investigated but not reported. Countries (in red) in which mupR *S. aureus* have been investigated and reported

An assessment of data on mupR *S. aureus* at the regional level is described as follows (Fig. 3).

### North Africa

Seventeen eligible studies were recorded from this region, including Egypt [28–31, 40, 52, 58], Tunisia [41–43, 59, 60], Libya [56, 57, 61], Algeria [47] and Morocco [62]. MupR *S. aureus* was reported in six studies conducted in two North African countries: Egypt [29–31, 52] and Libya [56, 57]. PCR detection of the *mupA* gene was performed in only two studies conducted in Egypt [30, 52]. In addition, one of the reports identified two *mupA* positive MRSA that exhibited LmupR [30]. MupR *S. aureus* was not detected in Tunisia [41–43, 59, 60], Algeria [47], and Morocco [62].

### West Africa

*S. aureus* resistance to mupirocin was investigated in Nigeria [35, 36, 44, 48, 49, 53, 63–66] and Ghana [54, 55, 67, 68]. Only two studies from Ghana reported on mupR *S. aureus* [54, 55]. In Nigeria, three studies (including two from only human sources and one from both animal and human samples, respectively) reported on *S. aureus* isolates that demonstrated HmupR [36, 44, 53].

### Central Africa

MupR *S. aureus* was not detected in studies conducted in Gabon [69], and São Tomé and Príncipe [70].

### East Africa

In this review, we identified four eligible studies conducted in Kenya [38, 71, 72] and Ethiopia [37]. A report on the role of cockroaches as potential vectors of foodborne pathogens in Ethiopia identified 17 mupR *S. aureus* isolates [37]. All the *S. aureus* isolates (one animal and two human studies) from Kenya were susceptible to mupirocin [38, 71, 72].

### Southern Africa

The six studies reported in this geographical area were from South Africa and consisted of two single centre studies [34, 46] and four multicenter studies [32, 33, 50, 51]. MupR *S. aureus* was identified in all the reports, while *mupA*-positive *S. aureus* isolates were noted in only two studies [33, 50].

### Prevalence of mupR *S. aureus* in Africa

**Fig. 4:**
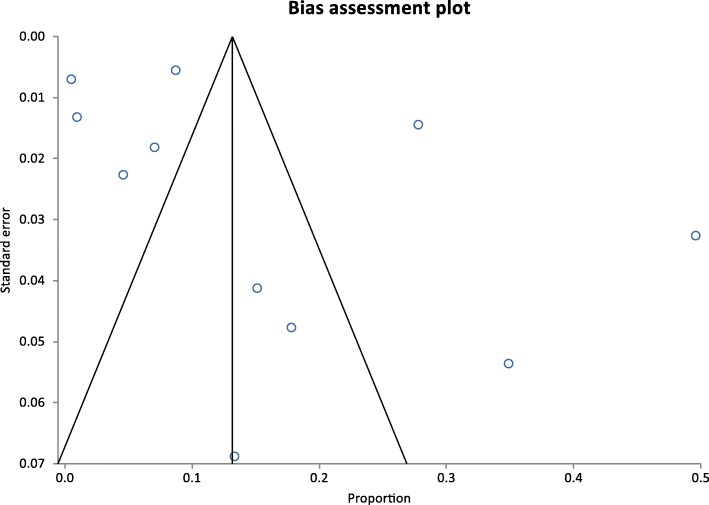
Bias assessment (Funnel) plot for studies assessing rates of mupirocin-resistant *Staphylococcus aureus* in Africa. Random effects (DerSimonian-Laird). Pooled proportion = 0.139303 (95% CI = 0.067511 to 0.23165). Bias indicators, Begg-Mazumdar: Kendall’s tau = 0.2 *P* = 0.4454, Egger: bias = 4.771137 (95% CI = −2.517874 to 12.060148) *P* = 0.1728, Harbord: bias = 2.014783 (92.5% CI = −5.90181 to 9.931377) *P* = 0.6208

**Fig. 5:**
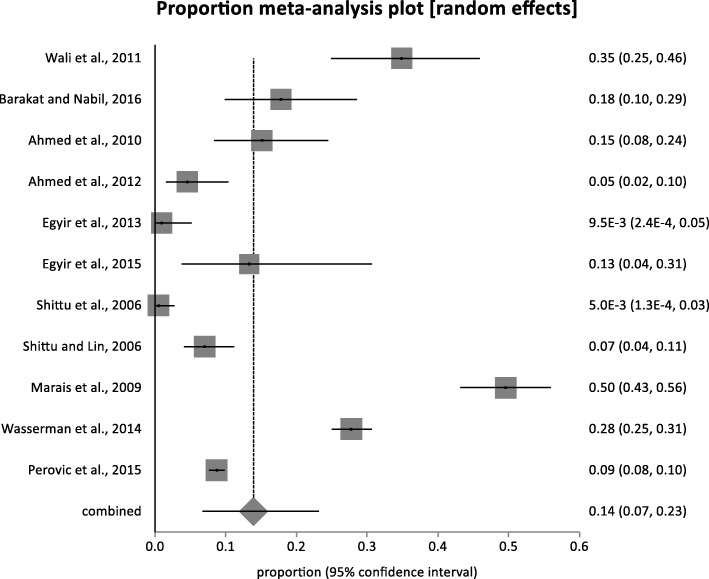
Pooled estimate of proportions (human studies) for mupirocin-resistant *Staphylococcus aureus* in Africa

The random-effects pooled prevalence of mupR *S. aureus* in Africa is 14% (95% CI =6.8 to 23.2%). This was calculated based on 11 heterogeneous human studies (Figs. 4 and 5) conducted in South Africa [32, 33, 50, 51], Ghana [54, 55], Egypt [30, 52], Libya [56, 57] and Nigeria [53]. In Africa, the proportion of *S. aureus* isolates with the *mupA* gene, and those that expressed LmupR and HmupR ranged between 0.5 and 8%, 4 and 47%, 0.5 and 38%, respectively. The frequency of mupR-MRSA isolates ranged between 5 and 50% (Table 3).

### Association of MupR *S. aureus* with mupirocin use in Africa

There is no data on the use of mupirocin as an agent for *S. aureus* decolonization and its association with mupR *S. aureus* in Africa.

### MupR *S. aureus* and biofilm production

A report from Egypt noted that mupR-MRSA were moderate to strong biofilm producers [52].

### MupR *S. aureus* and co-resistance to other antibiotics

In this systematic review, two studies (conducted in Egypt and South Africa) showed that mupR *S. aureus* was associated with multi-drug resistance [30, 33].

### Molecular characterization of mupR *S. aureus* in Africa

Only three studies provided molecular data on mupR *S. aureus* in Africa [45, 54, 55]. A report provided evidence of a 35 kb (non-conjugative) and 41.1 kb (conjugative) plasmid encoding *mupA* in *S. aureus* isolates from Nigeria and South Africa [45]. It also described an MRSA clone that demonstrated LmupR in South Africa. LmupR was also identified among MRSA isolates assigned with ST36, ST88, and ST789 in Ghana [55]. A cross-sectional *S. aureus* study identified a methicillin susceptible *S. aureus* (MSSA) strain with HmupR from a 51-year-old hospital staff in Ghana [54]. Molecular characterization indicated that the strain (*spa* type t4805) was PVL-positive.

## Discussion

This is the first systematic review on mupR *S. aureus* in Africa and clearly showed the paucity of data on the continent. Nevertheless, this study indicated a high prevalence ((14% (95% CI =6.8 to 23.2)) of mupR *S. aureus* in Africa. These observations support the need for mupR *S. aureus* surveillance data to provide information on its epidemiology and clinical significance in Africa. It is noteworthy that Google Scholar was valuable in the identification of several eligible studies [28–38]. We observed that 26% (11/43) of the eligible studies were identified from African journals which were not indexed in commonly used electronic databases. Google Scholar has been considered as a useful supplement with other electronic databases for systematic review search [73] including recent meta-analyses of published studies on *S. aureus* in Africa [74, 75].

The phenotypic methods for the screening and identification of mupR *S. aureus* include disc diffusion (two-disc strategy: 5 μg and 200 μg), agar dilution, broth micro-dilution and E-test [19]. In this study, the disk diffusion method and the CLSI (formerly NCCLS) guidelines were strategies mainly applied to detect mupR *S. aureus* in Africa. However, we observed certain inconsistencies [28, 29, 31, 33, 36, 40–49]. For instance, a number of studies [28, 29, 31, 33, 36, 40–42, 44–46] applied the disk diffusion method with the CLSI guidelines that had no breakpoint values for mupirocin. The 2017 CLSI guidelines recommend the use of the 200 μg disk to differentiate between HmupR and the absence of HmupR (i.e. no zone = HmupR; any zone = absence of HmupR) [76]. The 200 μg disk with a different breakpoint (Susceptible ≥30 mm, Resistance < 18 mm) is also endorsed for the differentiation between HmupR and the absence of HmupR in the latest versions (accessed 28th May, 2018) of the European Committee for Antimicrobial Susceptibility Testing (EUCAST) and Comité de l’antibiogramme de la Société Française de Microbiologie (CA-SFM) [77, 78]. The breakpoint values for the detection of LmupR and differentiation from HmupR are not provided in these documents (CA-SFM, CLSI, and EUCAST). Despite this limitation, the disk diffusion method in conjunction with any of these guidelines could at least be valuable for the preliminary screening and identification of HmupR *S. aureus* in Africa. MRSA decolonization failure is of clinical significance as it is often attributed to persistence or re-colonization associated with isolates exhibiting HmupR, while that of LmupR is not clear [7, 19, 79]. In this review, the prevalence of *S. aureus* that exhibited LmupR, HmupR and mupR-MRSA in Africa was predicated on a range of methods using different guidelines. We suggest that surveillance data from Africa is established on harmonized guidelines to enhance quality assurance and comparison at the continental and global level.

We noted a prevalence of mupR-MRSA ranging between 5 and 50% in Africa (Table 3). This is of serious concern. Specifically, the relationship between mupirocin resistance and MRSA has important consequences on infection control measures and effectiveness of decolonization strategies [8]. MupR-MRSA could limit the choices available for the control and prevention of healthcare-associated MRSA infections (7, 8). Therefore, surveillance studies are important to investigate the emergence and spread of mupirocin resistance in hospital settings in Africa. This is important among patients at high risk of MRSA infections, including patients in the dermatology, dialysis and the Intensive Care Units. In addition, there is the need for more data on the molecular characterization of mupR *S. aureus* in Africa [45, 54, 55]. For instance, whole genome sequencing (WGS) will assist in understanding the transmission dynamics of mupR *S. aureus* in Africa. Moreover, WGS data will allow comprehensive investigation of the genetic basis for LmupR mutation (which is largely due to V588F and V631F in the native gene (*ile*S)) and *mupB*-positive *S. aureus* in Africa.

Language bias was the main limitation of this systematic review as we did not include studies published in French, Portuguese, Arabic and Spanish.

## Conclusions

This study showed the need for more epidemiological data to understand the transmission, burden and risk factors associated with mupR *S. aureus* in Africa. In addition, there is a need for data on administration and use of mupirocin in community and hospital setting in Africa. This is important in antibiotic stewardship to mitigate the emergence and spread of mupR *S. aureus* in Africa. Finally, this systematic review highlighted the need for harmonized guidelines to facilitate the comparison of data on mupR *S. aureus* from Africa.

## Abbreviation

HmupR: High-level mupirocin resistance
LmupR: Low-level mupirocin resistance
MIC: Minimum inhibitory concentration
MRSA: Methicillin-resistant *Staphylococcus aureus*
MSSA: Methicillin-susceptible *S. aureus*
mupR: Mupirocin-resistant
PCR: Polymerase chain reaction
PVL: Panton Valentine Leucocidin
*S. aureus*: *Staphylococcus aureus*
ST: Sequence type
